# Supplementary material for: Evaluating the effectiveness of laser hair reduction using a home use laser in comparison to a Diode laser
Source: PLoS One. 2023 May 26;18(5):e0286162. doi: 10.1371/journal.pone.0286162 (PMC10218747; doi:10.1371/journal.pone.0286162)
Supplement: S1 Appendix — (DOCX) [file pone.0286162.s001.docx]

# APPENDIX A

**Basic requirements of a laser room**

- The room in which the laser is used needs to be labelled as a ‘controlled area’
- A sign must be fitted on the outside of the door of the room with the wording ‘Caution Controlled Area – Laser in Use’
- The door of the laser room should be made of opaque material to prevent transmission of laser light
- The lock should always be accessed from the inside in order the have uninterrupted exit in case of an emergency
- Laser machines require room temperatures between 18-22°C for normal functioning which requires the need for air-conditioning the laser room
- The laser room should be free of any reflective surfaces (mirrors/metal)
- Treatment kit for laser hair removal may include: razors, micro-pore tape, white marking pencil, soothing cream, electro gel, frozen ice/gel packs (if the laser machine does not have a built-in cooling device or cooling tip, laser specified goggles with the appropriate wavelength protection and surgical masks.
- A smoke evacuator is necessary to extract laser plumes.
