# Supplementary material for: Evaluating the effectiveness of laser hair reduction using a home use laser in comparison to a Diode laser
Source: PLoS One. 2023 May 26;18(5):e0286162. doi: 10.1371/journal.pone.0286162 (PMC10218747; doi:10.1371/journal.pone.0286162)
Supplement: Attachment — Submitted filename: Response to Reviewers.docx [file pone.0286162.s004.docx]

**Response to reviewers**

| **Reviewers comments and responses** | **Student response** | **Page** |
| --- | --- | --- |
| Ensure that your manuscript meets PLOS ONE’s style requirements, including those for file naming. | Thank you for your response. We have edited the article according to PLOS ONE style requirements to the best of our understanding of the instructions and used the links below.  <https://journals.plos.org/plosone/s/file?id=wjVg/PLOSOne_formatting_sample_main_body.pdf> and  <https://journals.plos.org/plosone/s/file?id=ba62/PLOSOne_formatting_sample_title_authors_affiliations.pdf> | 2,6,8,10,22 |
| Be clear about the difference between laser and IPL, the mechanisms of action to be further explained | Thank you for your response. The recommended changes were made and additional information has been added. | 2,3,4 |
| Limitations must be further discussed | Limitations has been discussed in the conclusion section | 17 |
| Additional references are required as mentioned | Additional references have been added in-text & reference page (Reference 12,15,20,24,25, 27, & 30) | 19 |
| Is the manuscript technically sound and do the data support the conclusion?  Reviewer 1: Yes | The data and conclusion have been revised and the data supports the conclusion | 17 |
| Has the statistical analysis been performed appropriately and rigorously?  Reviewer 1: I don’t know | Statistical analyses was performed appropriately by a statistician using the latest Statistical Package for social science (SPSS) version 27 | 8 |
| Have the authors made all data underlying the finding in their manuscript fully available?  Reviewer 1: Yes | Yes |  |
| Is the manuscript presented in an intelligible fashion and written in standard English?  Reviewer 1 Yes | Yes |  |
| The title is inaccurate and misleading. It should read: “Evaluating the effectiveness of laser hair reduction using a home-use ‘intense pulsed light’ device in comparison with a professional diode laser” | Thank you for your response.  We have accepted the change in the heading and corrected the title of the manuscript as follows: Evaluating the effectiveness of laser hair reduction using a home-use ‘intense pulsed light’ device in comparison with a professional diode laser. | 1 |
| There must be a distinction between 'laser' and 'intense pulsed light' as they are radically different technologies and this must be maintained throughout the article to avoid misunderstanding.  The mode of action in the introduction needs to be clarified and expanded. | Thank you for your response.  We have accepted the changes between laser & IPL.  We have expanded on the mode of action | 2,3,4 |
| Retinal injury is not the only risk, consider the pigmented iris! In the discussion, there is insufficient reference to published data on safety of home-use IPL devices | Thank you for your response.  We have accepted the additional information added another review. | 15 |
| The statements about loss of skin contact and light spillage require clarification and expansion | Thank you for your response.  We have made the additional changes and added information. | 16 |
| The text about US FDA 510k pre-marketing clearance of the devices used in this study relates only to permission to sell a product in the USA based on predicate information and has no relevance to this study. Reference FDA clearances is correctly used in the conclusions. | Thank you for your comment.  It has been removed. | 16 |
| The Conclusions need to be revised and expanded to include limitations of this study e.g. small sample size, need for more randomised, controlled studies, etc . | Thank you for your response.  The conclusion includes limitations | 17 |
| Several important references are missing including recent publications in international peer-reviewed journals | Additional references have been added in-text & in references | 19 |
