# Supplementary material for: Evaluating the effectiveness of laser hair reduction using a home use laser in comparison to a Diode laser
Source: PLoS One. 2023 May 26;18(5):e0286162. doi: 10.1371/journal.pone.0286162 (PMC10218747; doi:10.1371/journal.pone.0286162)
Supplement: Attachment — Submitted filename: 2 Response to reviewers.docx [file pone.0286162.s005.docx]

**Response to reviewers**

| **Reviewers comments** | **Responses** | **Pages** |
| --- | --- | --- |
| You have changed the abstract in the submission system but have not changed the text in the paper. Perhaps you submitted the wrong version? You must replace all instances of "home use laser" with "home use IPL device"   Please submit a version with all the changes highlighted | Thank you for your response.  The article has been edited and changed all “home-use laser” to “home-use IPL device” and “home-use IPL” | 3,5,6,7,10,13,14,16 &17 |
